# Supplementary material for: Lead exposure is associated with functional and microstructural changes in the healthy human brain
Source: Commun Biol. 2021 Jul 26;4:912. doi: 10.1038/s42003-021-02435-0 (PMC8313694; doi:10.1038/s42003-021-02435-0)
Supplement: Supplementary file 3 — Reporting Summary [file 42003_2021_2435_MOESM3_ESM.pdf]

Corresponding author(s): Hikaru Takeuchi

Last updated by author(s): Jun 19, 2021

Reporting Summary

Nature Research wishes to improve the reproducibility of the work that we publish. This form provides structure for consistency and transparency in reporting. For further information on Nature Research policies, see our Editorial Policies and the Editorial Policy Checklist.

Statistics

For all statistical analyses, confirm that the following items are present in the figure legend, table legend, main text, or Methods section.

n/a	Confirmed	
☐	☒	The exact sample size (*n*) for each experimental group/condition, given as a discrete number and unit of measurement
☒	☐	A statement on whether measurements were taken from distinct samples or whether the same sample was measured repeatedly
☐	☒	The statistical test(s) used AND whether they are one- or two-sided<br>*Only common tests should be described solely by name; describe more complex techniques in the Methods section.*
☐	☒	A description of all covariates tested
☐	☒	A description of any assumptions or corrections, such as tests of normality and adjustment for multiple comparisons
☐	☒	A full description of the statistical parameters including central tendency (e.g. means) or other basic estimates (e.g. regression coefficient) AND variation (e.g. standard deviation) or associated estimates of uncertainty (e.g. confidence intervals)
☐	☒	For null hypothesis testing, the test statistic (e.g. *F*, *t*, *r*) with confidence intervals, effect sizes, degrees of freedom and *P* value noted<br>*Give P values as exact values whenever suitable.*
☒	☐	For Bayesian analysis, information on the choice of priors and Markov chain Monte Carlo settings
☒	☐	For hierarchical and complex designs, identification of the appropriate level for tests and full reporting of outcomes
☐	☒	Estimates of effect sizes (e.g. Cohen's *d*, Pearson's *r*), indicating how they were calculated

Our web collection on statistics for biologists contains articles on many of the points above.

Software and code

Policy information about availability of computer code

Data collection	No software for data collection.
Data analysis	Behavioral data were analyzed using R software version 4.0.1. P-values of behavioral data were assessed by permutation (5,000 iterations) based on multiple regression analyses using the lmPerm package 61 and R software.<br>SPM8 was used for preprocessing and statistical analyses of whole brain neuroimaging data.<br>For the cross-sectional whole brain analyses, multiple comparison corrections were performed using threshold-free cluster enhancement (TFCE) 52 with randomized (5,000 permutations) nonparametric testing using the TFCE toolbox (http://dbm.neuro.uni-jena.de/tfce/).

For manuscripts utilizing custom algorithms or software that are central to the research but not yet described in published literature, software must be made available to editors and reviewers. We strongly encourage code deposition in a community repository (e.g. GitHub). See the Nature Research guidelines for submitting code & software for further information.

Data

Policy information about availability of data

All manuscripts must include a data availability statement. This statement should provide the following information, where applicable:

- Accession codes, unique identifiers, or web links for publicly available datasets
- A list of figures that have associated raw data
- A description of any restrictions on data availability

All of the experimental data obtained in this study will be available for those approved in the Ethics Committee of Tohoku University's medical faculty. All data sharing activities must be first approved by the Ethics Committee of Tohoku University's medical faculty.

Field-specific reporting

Please select the one below that is the best fit for your research. If you are not sure, read the appropriate sections before making your selection.

- [x] Life sciences
- [ ] Behavioural & social sciences
- [ ] Ecological, evolutionary & environmental sciences

For a reference copy of the document with all sections, see nature.com/documents/nr-reporting-summary-flat.pdf

Life sciences study design

All studies must disclose on these points even when the disclosure is negative.

Sample size	The present study is part of an ongoing project that aims to investigate associations between brain imaging, cognitive functions, and aging and included 920 healthy right-handed individuals (561 men and 359 women) from whom the data necessary for whole-brain analyses involving lead levels were collected. The mean subject age was 20.7 years (standard deviation, 1.8; age range: 18–27 years).
Data exclusions	Data analyses of each measure was conducted using the data of the sample from whom all the dependent and independent measures were properly obtained.
Replication	There are no replication analyses.
Randomization	Randomization procedures are not irrelevant in this study.
Blinding	Blinding procedures are not irrelevant in this study.

Reporting for specific materials, systems and methods

We require information from authors about some types of materials, experimental systems and methods used in many studies. Here, indicate whether each material, system or method listed is relevant to your study. If you are not sure if a list item applies to your research, read the appropriate section before selecting a response.

Materials & experimental systems

n/a	Involved in the study	
x		Antibodies
x		Eukaryotic cell lines
x		Palaeontology and archaeology
x		Animals and other organisms
	x	Human research participants
x		Clinical data
x		Dual use research of concern

Methods

n/a	Involved in the study	
x		ChIP-seq
x		Flow cytometry
	x	MRI-based neuroimaging

Human research participants

Policy information about studies involving human research participants

Population characteristics	The present study is part of an ongoing project that aims to investigate associations between brain imaging, cognitive functions, and aging and included 920 healthy right-handed individuals (561 men and 359 women) from whom the data necessary for whole-brain analyses involving lead levels were collected. The mean subject age was 20.7 years (standard deviation, 1.8; age range: 18–27 years).<br><br>All subjects were either undergraduate students, graduate students or fresh graduates. All subjects had normal vision and none had neurological or psychiatric illnesses. Handedness was evaluated using the Edinburgh Handedness Inventory 2.
Recruitment	Details of recruitment and exclusion criteria of subjects<br>They were recruited using advertisements on bulletin boards at Tohoku University or via email introducing the study. These advertisements and emails specified the unacceptable conditions in individuals with regard to participation in the study such as handedness, the existence of metal in and around the body, claustrophobia, the use of certain drugs, a history of certain psychiatric and neurological diseases, and previous participation in related experiments.<br>A history of psychiatric and neurological diseases and/or recent drug use was assessed using our laboratory's routine questionnaire, in which each subject answered questions related to their current or previous experiences of any of the listed diseases and listed drugs that they had recently taken. Drug screening was performed to confirm that the subjects were not taking any illegal psychostimulants or antipsychotic drugs, which was one of the exclusion criteria used during the course of the recruitment. Subjects with exclusion criteria should have been excluded before they came to the lab, but if they came for some reason, they had to go back once it was found that they met an exclusion criterion. Consequently, none had a history of neurological or psychiatric illness. In the course of this experiment, the scans were checked for obvious brain lesions and tumors, but there were no subjects having such obvious lesions or tumors.<br>These descriptions are mostly obtained from our previously published work 4.
Ethics oversight	Approval for these experiments was obtained from the Institutional Review Board of Tohoku University.

Note that full information on the approval of the study protocol must also be provided in the manuscript.

Magnetic resonance imaging

Experimental design

Design type	Cross-sectional observation study
Design specifications	We investigated the associations of hair lead levels with cognitive measures, brain activity, FA of the white matter, and MD of gray and white matter areas in a large cohort of typically developing young adults.
Behavioral performance measures	Psychological measures<br>Following neuropsychological testing, several questionnaires were administered to the participants. These tests were chosen because of the known effects of lead on a wide range of cognitive functions, dopaminergic mechanisms, attention deficit, and several mood states, as described earlier. The test descriptions in this subsection are largely reproduced from our previous studies 36. For full details, see Supplemental Methods.<br>[A] RAPM 54 is a non-verbal reasoning task and representative measure of general intelligence. More details of this task are available in our previous study 55.<br>[B] The Tanaka B-type intelligence test (TBIT) 56 type 3B (TBIT) is a non-verbal mass intelligence test used for third-year junior high school and older examinees in Japan. Rather than using story problems, the test uses figures, single numbers, and letters as stimuli. The subjects must solve as many problems as possible within a certain time (a few minutes) in all subtests, which means these problems are complex cognitive speed tasks. More details on the TBIT are available in our previous study 57.<br>[C] Two arithmetic tasks measured performance in two forms of one-digit times one-digit multiplication problems (i.e., a simple arithmetic task with numbers between 2 and 9) and two forms of two-digit times two-digit multiplication problems (i.e., a more complex arithmetic task with numbers between 11 and 19). The subjects were asked to solve as many questions as possible in simple and complex arithmetic tasks within 30 and 60 s, respectively.<br>[D] Hakoda's version of the Stroop task 58 was used to measure response inhibition and impulsivity. This version of the matching-type Stroop task requires subjects to check whether their chosen answers are correct, unlike the traditional oral-naming Stroop task. The test consists of two control tasks, namely, Word-Color and Color-Word tasks, a Stroop task, and a reverse Stroop task. Reverse Stroop and Stroop interference rates were calculated from the scores obtained from these tasks. Details of this test are provided in our previous study 59.<br>[E] The reading comprehension task used in this study was developed by Kondo et al. 60. More details on this test, such as how it was developed and its validity, are provided by Kondo et al. 60 and our previous study 61.<br>[F] S-A creativity test. Creativity as divergent thinking was measured using the S-A creativity test 62. More details are available in our previous study 55.<br>[G] A (computerized) digit-span task, which is a working memory task for details, see 63.<br>[H] The motivational state of the day for each subject was measured using the Vigor subscale of a shortened Japanese version 64 of the Profile of Mood States psychological rating scale 65.<br>[I] The Japanese version 66 of the Temperament Character Inventory 67 was used to measure novelty seeking. A subscale of this measure, that is, impulsiveness, was also used to measure impulsivity.<br>[J] The Japanese version of the NEO Five-Factor Inventory (NEO-FFI) was used to measure extraversion 68.

[K] The cognitive reflectivity–impulsiveness questionnaire 69 was used to assess individual differences in reflectivity and impulsivity 70.
[L] The External-Preoccupation Scale 71 was used to measure the maintenance of external focus on a specific object. Data for this scale were collected only from a subset of the subjects (i.e., 678 successfully genotyped subjects).
[M] The Japanese version 72 of the Beck Depression Inventory (BDI) 73 was used to measure the current state of depression.

Acquisition

Imaging type(s)

Diffusion-weighted data and functional activation data

Field strength

3T

Sequence & imaging parameters

Diffusion-weighted data were acquired using a spin-echo EPI sequence (TR = 10293 ms, TE = 55 ms, FOV = 22.4 cm, 2×2×2 mm3 voxels, 60 slices, SENSE reduction factor = 2, number of acquisitions = 1). The diffusion weighting was isotropically distributed along 32 directions (b value = 1,000 s/mm2). In addition, three images with no diffusion weighting (b value = 0 s/mm2) (b = 0 images) were acquired using a spin-echo EPI sequence (TR = 10293 ms, TE = 55 ms, FOV = 22.4 cm, 2 2 2 mm3 voxels, 60 slices). FA and MD maps were calculated from the images collected using a commercially available diffusion tensor analysis package on the MR console. The descriptions in this subsection are mostly reproduced from a previous study using similar methods 27.
Forty-two transaxial gradient-echo images (TR = 2.5 s, TE = 30 ms, flip angle = 90°, slice thickness = 3 mm, FOV = 192 mm, matrix = 64 × 64) covering the entire brain were acquired using an echo planar sequence. A total of 174 functional volumes were obtained for the n-back sessions. The consideration and exclusion of movement effects during fMRI analyses are described in Supplemental Methods.

Area of acquisition

Whole-brain

Diffusion MRI ☒ Used ☐ Not used

Parameters: Described in sequence & imaging parameters

Preprocessing

Preprocessing software

SPM8

Normalization

Preprocessing of imaging data
Preprocessing and analysis of functional activation data were performed using SPM8 implemented in MATLAB. Descriptions in this subsection were mostly reproduced from a previous study using similar methods 26. Before analysis, individual BOLD images were re-aligned and re-sliced to the mean BOLD image, and corrected for slice timing. The mean BOLD image was then realigned to the mean b = 0 image together with the slice-timing-corrected images, as described previously 37. Because the mean b = 0 image was aligned with the FA image and MD map, the BOLD image, b = 0 image, FA image, and MD map were all aligned. Subsequently, using a previously validated two-step segmentation algorithm of diffusion images and diffeomorphic anatomical registration through an exponentiated lie algebra (DARTEL)-based registration process 29, all images—including gray matter segments [regional gray matter density (rGMD) map], white matter segments [regional white matter density (rWMD) map], and cerebrospinal fluid (CSF) segments [regional CSF density (rCSFD) map] of the diffusion images—were normalized.
The details of these procedures, which were also described in our previous study 29, are as follows. Using the new segmentation algorithm implemented in SPM8, FA images of each individual were segmented into six tissues (first new segmentation). The default parameters and tissue probability maps were used in this process, except that affine regularization was performed using the International Consortium for Brain Mapping template for East Asian brains and the sampling distance (approximate distance between sampled points when estimating the model parameters) was 2 mm. We then synthesized the FA image and MD map. In the synthesized image, the area with a WM tissue probability >0.5 in the abovementioned new segmentation process was the FA image multiplied by −1 (hence, the synthesized image shows very clear contrast between WM and other tissues); the remaining area is the MD map (for details of this procedure, see below). The synthesized image from each individual was then segmented using the new segmentation algorithm implemented in SPM8 with the same parameters as above (second new segmentation). This two-step segmentation process was adopted because the FA image has a relatively clear contrast between GM and WM, as well as between WM and CSF, and the first new segmentation step can segment WM from other tissues. On the other hand, the MD map has clear contrast between GM and CSF and the second new segmentation can segment GM. Since the MD map alone lacks clear contrast between WM and GM, we must use a synthesized image (and the two-step segmentation process).
We then proceeded to the DARTEL registration process implemented in SPM8. We used the DARTEL import image of the GM tissue probability map produced in the second new segmentation process as the GM input for the DARTEL process. The WM input for the DARTEL process was created as follows. First, the raw FA image was multiplied by the WM tissue probability map from the second new segmentation process within the areas with a WM probability >0.5 (signals from other areas were set to 0). Next, the FA image * WM tissue probability map was coregistered and resliced to the DARTEL import WM tissue probability image from the second segmentation. The template for the DARTEL procedures was created using imaging data from 63 subjects who participated in the experiment in our lab 37 and were included in the present study (meaning that they have the same characteristics as the subjects in this study). The first reason why we created the DARTEL template from the images of a subset of all subjects (63 subjects) and not from all subjects is because this is a large sample for creating a template compared to previous studies and thus cannot be considered problematic. The second reason is that the project in which the subjects participated is ongoing, and the DARTEL processes—especially our processes—require vast amounts of time and the resultant images require large storage resources; thus, we cannot reprocess the images of all subjects and add

newer images whenever we change the number of subjects. Next, using this existing template, the DARTEL procedures were performed for all subjects in this study. In these procedures, the parameters were changed as follows to improve accuracy. The number of Gauss–Newton iterations performed within each outer iteration was set to 10 and, in each outer iteration, we used 8-fold more timepoints to solve the partial differential equations than the default values. The number of cycles used by the full multi-grid matrix solver was set to 8. The number of relaxation iterations performed in each multi-grid cycle was also set to 8. The resultant synthesized images were spatially normalized to MNI space. Using these parameters, the raw FA map, rGMD, and rWMD map from the abovementioned second new segmentation process were normalized to give images with 1.5 1.5 1.5 mm3 voxels. The FA image * WM tissue probability map was used in the DARTEL procedures because it includes different signal intensities within WM tissues and the normalization procedure can take advantage of intensity differences to adjust the image to the template from the perspective of the outer edge of the tissue and within the WM tissue. No modulation was performed in the normalization procedure.

The voxel size of the normalized FA images, MD images, and segmented images was 1.5 1.5 1.5 mm3. The voxel size of the normalized BOLD images was 3 3 3 mm3.

Next, we created average images of normalized rGMD and rWMD images from the normalized rGMD and rWMD images from the subset of the entire sample (63 subjects) 29. From the average image of normalized WM segmentation images from the 63 subjects mentioned above, we created mask image consisting of voxels with a WM signal intensity > 0.99. We then applied this mask image to the normalized FA image, thereby only retaining areas highly likely to be white matter. These images were smoothed (6 mm full-width half-maximum) and carried through to the second-level analyses of FA. As described previously 29, through application of the mask, images unlikely to be WM or border areas between WM and other tissues were removed. The FA images were not affected by signals from tissues other than WM even after smoothing. This is important considering that, in these areas, WM volume and FA are highly correlated 38 and the FA map supposedly reflects the extent of WM. Further, differences in WMC compared with other tissues among individuals can be ignored after application of this mask because, within the masks, all voxels show very high white mater probability. For validation of these preprocessing methods and comparison with other methods, see the supplementary online material of our previous study 29.

Through these procedures, we believe that we successfully mitigated or removed the problems of voxel-based analysis of FA analysis raised by Smith et al 39. These problems include (a) misalignment within white matter tissue (addressed by new segmentation processes and DARTEL processes that utilized difference in signal distribution within white matter using the FA signal) and (b) the effects of different tissue types and partial volume effects (addressed by new segmentation processes, the DARTEL processes, and application of the mask confined to images highly likely to be white matter (in the case of MD maps, white matter or gray matter)). Through these methods, the white matter of DTI images as well as the gray matter areas of DTI images become available for analysis. We avoided co-registration of DTI images to T1 weighted structural images because the shapes differ due to the unignorable distortion of EPI images in 3T MRI.

Normalization template

Described in "Normalization space". The standardized space is MNI305.

Noise and artifact removal

Diffusion data. The acquisitions for phase correction and signal stabilization were not used as reconstructed images. MD and FA maps were calculated from the collected images using a commercially-available diffusion tensor analysis package on the MR console. This method has been used in many of our previous studies 28-32. The image-generated results were congruent with those of previous studies using other methods 33, 34, suggesting the validity of this method. The procedures involved correction for motion and distortion caused by eddy currents. Calculations were performed according to a previously proposed method 35. Descriptions in this subsection were mostly reproduced from a previous study using similar methods 27.

Functional data. The design matrix weighted each raw image according to its overall variability to reduce the impact of movement artifacts 58.

Volume censoring

Visual inspection

Statistical modeling & inference

Model type and settings

Statistical analyses of non-whole-brain analyses

Behavioral data were analyzed using R software version 4.0.1 59, and the associations of hair lead levels with psychological outcome measures were tested using multiple regression analyses. A total of 16 cognitive variables were included as dependent, as presented in Table 1. The independent variables included sex, age, self-reported height, self-reported weight, body mass index (calculated from self-reported height and weight), annual family income, parents' highest educational qualifications (measured as reported in 60), and hair lead levels. P-values were assessed by permutation (5,000 iterations) based on multiple regression analyses using the lmPerm package 61 and R software.

The expression for each test is as follows.

```
Result_x <- lmp(Test_x ~ sex + age + height + weight + BMI + parents_education_level + family_income + hair_lead_level,
datasetname_y, seqs = TRUE)
summary(Result_x)
```

Permutation analyses were conducted nine times (conducting 9 times lead to more stable results and the number was chosen empirically) for each cognitive measure, and the median p-value was used for analyses.

Whole-brain statistical analysis

We investigated whether the imaging measures are associated with individual differences in hair lead. Whole-brain multiple regression analyses were performed using SPM8.

The covariates used in the FA and MD analyses were identical to those applied for psychological analyses; volume-level mean frame-wise displacement during the diffusion scan was also added as a covariate for these analyses. FA analysis was performed within the white matter mask created above, and MD analysis was performed within the gray matter + white matter mask.

In the fMRI analyses, the maps of dependent variables were beta estimate images of 2-back > 0-back contrast. The covariates used for this analysis included those used in the psychological analyses, as well as accuracies and reaction times in the 0-back and 2-back tasks and volume-level mean frame-wise displacement during the scan for the n-back task 63.

Effect(s) tested

individual differences in hair lead level (logarithm)

Specify type of analysis: ☒ Whole brain ☐ ROI-based ☐ Both

Statistic type for inference
(See Eklund et al. 2016)

Described in model type and settings

Correction

Statistical analyses of non-whole-brain analyses
For all analyses, results with a threshold of $P < 0.05$ (two-sided) after correcting for the false discovery rate (FDR) using a two-stage sharpened method 62 were considered statistically significant.

Whole-brain statistical analysis
Correction for multiple comparisons was performed using threshold-free cluster enhancement (TFCE) 64 with randomized (5,000 permutations) nonparametric testing using the TFCE toolbox (http://dbm.neuro.uni-jena.de/tfce/). The family-wise error (FWE) threshold was corrected at $p < 0.025$.

Models & analysis

n/a	Involved in the study
☒	☐ Functional and/or effective connectivity
☒	☐ Graph analysis
☒	☐ Multivariate modeling or predictive analysis
