# Supplementary material for: Lead exposure is associated with functional and microstructural changes in the healthy human brain
Source: Commun Biol. 2021 Jul 26;4:912. doi: 10.1038/s42003-021-02435-0 (PMC8313694; doi:10.1038/s42003-021-02435-0)
Supplement: Supplementary file 4 — Description of Additional Supplementary Files [file 42003_2021_2435_MOESM4_ESM.pdf]

Description of Additional Supplementary Files

File name: Supplementary Data 1

Description: Dependent and independent variables of multiple regression analyses behind residual plots of Fig. 1 – Fig. 4.
