# Supplementary material for: Lead exposure is associated with functional and microstructural changes in the healthy human brain
Source: Commun Biol. 2021 Jul 26;4:912. doi: 10.1038/s42003-021-02435-0 (PMC8313694; doi:10.1038/s42003-021-02435-0)
Supplement: Supplementary file 5 — Supplementary Information [file 42003_2021_2435_MOESM5_ESM.pdf]

Supplementary Information

Supplemental Fig. 1.

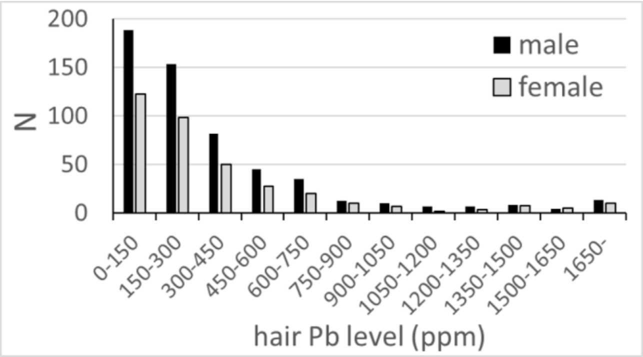

Supplemental Fig. 1. Histograms showing raw head lead levels in male and female subjects.

Supplemental Fig. 2.

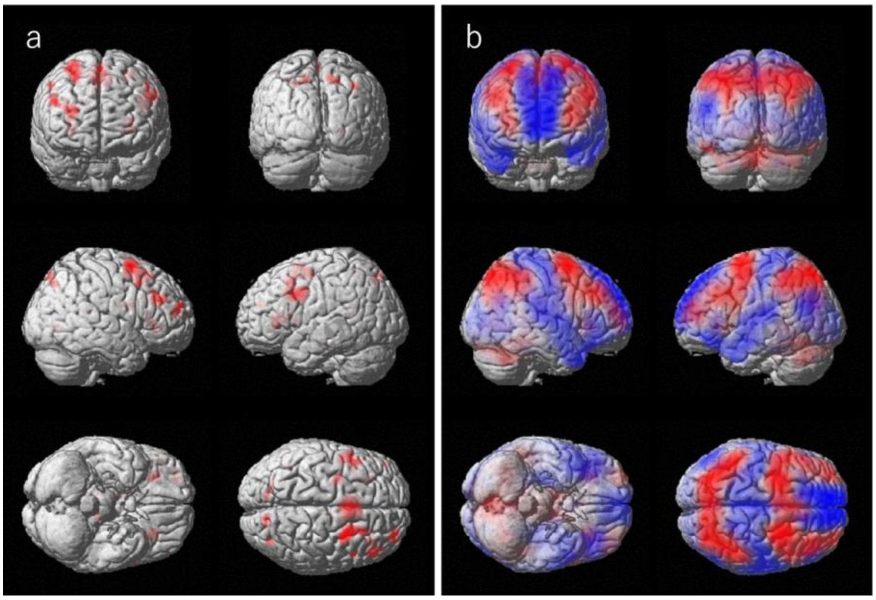

Supplemental Fig. 2. Tendencies of positive associations between increases in brain activity and hair lead levels (N = 892). (a) Regions with a tendency of positive associations between the brain activity of the contrast (2 back – 0-back) and hair lead levels were overlaid on a “render” image from SPM8. Results were obtained using a threshold of $p < 0.001$, uncorrected. Strong positive associations were found in areas showing an increase in brain activity in widespread areas of the fronto-parietal network demonstrating increased brain activation during the working memory test. (b) Areas of significant activation for the contrast (2-back–0-back) (red) and significant deactivation for the contrast (2-back–0-back) (green) were overlaid on a “render” image from SPM8. Results were obtained using a threshold of $P < 0.05$, corrected for FDR for the entire sample (N = 1235) with effective N-back fMRI activation data for this project [1].

Supplementary References

1. Takeuchi H, Taki Y, Nouchi R, Yokoyama R, Kotozaki Y, Nakagawa S *et al.* General intelligence is associated with working memory-related brain activity: new evidence from a large sample study. *Brain Struct Funct* 2018; **223**(9)**:** 4243-4258.
